# Exploring the use of chamomile (*Matricaria chamomilla* L.) bioactive compounds to control flixweed (*Descurainia sophia* L.) in bread wheat (*Triticum aestivum* L.): Implication for reducing chemical herbicide pollution

**DOI:** 10.1016/j.sjbs.2022.103421

**Published:** 2022-08-27

**Authors:** Elham Madadi, Sina Fallah, Amir Sadeghpour, Hossien Barani-Beiranvand

**Affiliations:** a Student of Crop Ecology, Faculty of Agriculture, Shahrekord University, Shahrekord, Iran; b Professor of Agronomy, Faculty of Agriculture, Shahrekord University, Shahrekord, Iran; c School of Agricultural Sciences, Southern Illinois University, Carbondale, USA; d Assistant Professor, Department of Biology, Islamic Azad University of Najafabad, Najafabad, Iran

**Keywords:** Bioherbicide, Cell viability, Mitotic index, Phenol, Seedling, Terpenoids

## Abstract

Intensive chemical herbicide use has resulted in human health and environmental issues. This study evaluated the phytotoxic potential of chamomile extract as a bioherbicide to minimize chemical herbicide use in wheat production. Treatments including four concentrations (0, 50, 100, and 150 mL/L) of three different chamomile plant parts (root, shoot, and root + shoot) extracts were applied to flixweed as a major weed in wheat production. Except for 50 mL/L of root extract, other concentrations of chamomile extracts decreased the germination rate of flixweed. Germnaiton rate of wheat increased with chamomile extracts except at 150 mL/L concentration of shoot extract at which the germination rate of flixweed and wheat reduced by 71.7 and 35.4%, respectively, compared to respective controls. Compared to wheat, malondialdehyde and proline in flixweed were increased fivefold in flixweed and compared to the control, ranged from 84–473 and 240–1422%, respectively. Chamomile extract also declined cell viability much quicker in flixweed than in wheat reflecting on greater inhibitory effect for flixweed control. Chamomile shoot extract reduced seedling weight and vigor index of flixweed by 63.75 and 59.4%, respectively, compared to the respective control. Results of liquid chromatography mass spectrometry of chamomile extract indicated polyphenols, flavonoids, terpenoids, and bioactive phenolic coumarins, glycosylated derivatives, quercetin and its derivatives, herniarin, umbelliferone, P-cymene, chamazulene, farnesol, amitrole, 1,8-cineole, and limonene were effective in inhibiting the germination and growth of flixweed. We concluded that 150 mg/L of chamomile shoot extract could be used as a bioherbicide to sustainably suppress flixweed in wheat production.

## Highlights

• The investigation of the bioactive effects of chamomile bioactive on weed is the first report.
• Chamomile can be cultivated as a pre-planting in chamomile-wheat rotation.
• Chamomile can be introduced as a viable candidate for the production of bioherbicide.
• Chamomile allelochemicals has suppressive effects on weeds both at the cellular up to organ levels.

## Graphical abstract

**Figure:**
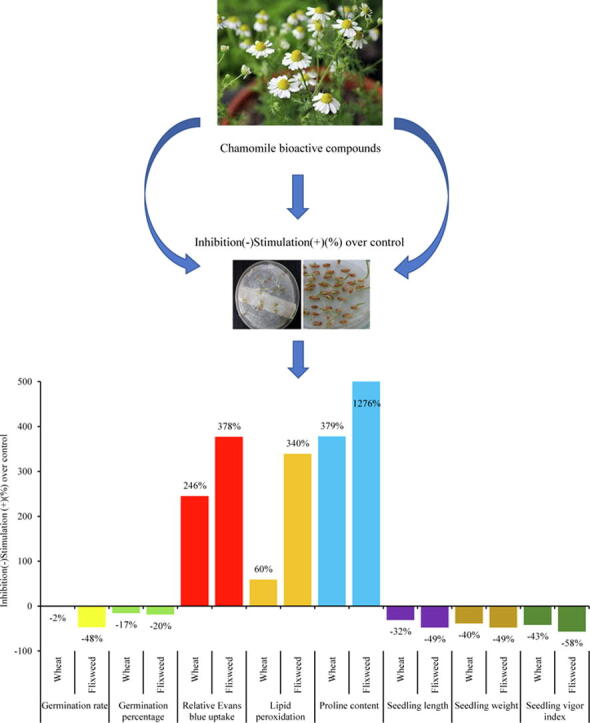

## 1 Introduction

Wheat is a major crop in Iran and many other countries (FAO, 2019). High production of this crop in Iran and the world (763.5 and 14.5 million tons; FAO, 2019) indicates the importance of wheat as a source of food for many countries, including Iran. Weeds are a major limiting factor in wheat production due to their direct competition with wheat for resources, including light, nutrients, and soil moisture (Colbach et al., 2017). In addition, weeds can serve as hosts for pathogens and pests that constantly interfere with crop growth (Colbach et al., 2017). Therefore, managing weeds is critical to maximizing wheat yield and quality.

In agroecosystems, weeds at high density during the critical stages of cash crop growth reduces cash crop yield potential (Chauhan, 2020, Cordeau et al., 2021). Herbicides are effective tools for controlling weeds in the cropping system. However, long-term use of chemical herbicides not only harmfully affects human and environmental health but also increases herbicide-resistant weeds (Scavo and Mauromicale, 2021). To avoid these effects, exploring allelochemicals from potent allelopathic plants has become critical in the search for weed control alternatives to synthetic herbicides (Anwar et al., 2021). These allelochemicals gained from plants belong to diverse chemical groups like fatty acids, phenolics, alcohols, steroids, flavonoids, and terpenoids (Singh et al., 2011). Most allelochemicals are eco-friendly due to their structural organization, short half-life in soils, and synergistic action when released into the environment (Kyaw et al., 2022). There are different mechanisms by which allelopathy can be exploited to manage weeds: cover cropping, crop rotation with allelopathic species, intercropping, and plant extracts (Scavo and Mauromicale, 2021). An increase in reactive oxygen species production (ROS), alteration of membrane permeability and cell structure, change of photosynthesis and respiration, inhibition and/or reduction of seed germination and seedling growth, are documented as effects of plant extract (Radhakrishnan et al., 2018).

Many medicinal plants have been reported to have allelopathic compounds, and their extracts have been used to suppress weeds (Sothearith et al., 2021, Sothearith et al., 2021; Kyaw et al., 2022). For example, it is reported that the extract of *Ocimum gratissimum*, *Chromolaena odorata*, *Ageratum conyzoides*, and *Eclipta alba* have allopathic potential (Appiah et al., 2017). In another experiment, the phytotoxic potential of 105 medicinal herbs in the arid/semiarid coastal areas of Saudi Arabia and Pakistan were investigated (Qasim et al., 2019). The ingredients of the medicinal herbs had inhibitory effects on the seed germination and growth of other plants (Badmus and Afolayan, 2012), and all these plants can be used for weed control (Kato-Noguchi, 2003, Fernandez-Aparicio et al., 2008). The German chamomile (*M. chamomilla*) is a medicinal plant whose extract contains 120 types of chemical compounds, including chamazulene, coumarins and flavonoids. The most important active ingredients of these compounds are chamazulene, apigenin and alpha-bisabolol (McKay and Blumberg, 2006, Singh et al., 2011). The chamomile extract compounds have antibacterial, anti-inflammatory and antioxidant activities (Srivastava et al., 2010, Wu et al., 2011). This plant has high flavonoids, which are effective antioxidants for scavenging oxygenated radicals (McKay and Blumberg, 2006, Abdoul-Latif et al., 2011).

Flixweed (*D. sophia*) is an herbaceous and shrub plant that grows in autumn as a companion weed with wheat, rapeseed (*Brassica napus*), and alfalfa (*Medicago sativa* L). Flixweed germinates simultaneously with wheat but grows faster, matures earlier, and produces a lot of seeds that can be easily incorporated into the soil seed bank. Growers use herbicides including 2,4-D + MPCA, MCPA-Na + carfentrazone-ethyl, and florasulam to control flixweed in wheat (Wang et al., 2021). These herbicides have a relatively long shelf life, are important soil and water pollutants, and are very toxic to plants, animals, and humans. Therefore, finding an effective strategy to control flixweed in wheat production could significantly impact wheat growth and production and reduce uncertainties regarding food security. To the best of our knowledge, this is the first attempt to evaluate the chamomile allelopathic effect on flixweed in wheat production. Accordingly, we hypothesized that: (1) camomile extract suppresses the growth of flixweed seedlings, (2) the inhibition of different organs extract is different, and (3) chamomile bioactive compounds is a selective herbicide for wheat.

## 2 Materials and methods

### 2.1 Experimental site and design

Each experiment (wheat and flixweed) was conducted in a completely randomized design with three replicates under growth chamber conditions in 2020 in the Faculty of Agriculture, Shahrekord University. Each experiment consisted of two factors with a factorial arrangement of 3 × 4. Plant organ extracts (1-root, 2-shoot, and 3-whole plant (root plus shoot)) of chamomile and extract concentrations (0, 50, 100, and 150 mL/L) were the main factors.

### 2.2 Experimental set up

The seeds of flixweed were collected from wheat fields in the Shahrekord region (32°21′N, 50°49′E with 2050 m asl) on 10 June 2020. Then, the tetrazolium test was used to ensure the viability of the weed seeds. For this purpose, the weeds were soaked in a 0.1% 2,3,5-triphenyl tetrazolium chloride solution. For seed dormancy breaking, seeds were soaked in 1000 mg/L potassium nitrate solution for 24 h and then washed with distilled water (Baskin and Baskin, 2014). Wheat seeds (cv. Pishgam) were obtained from Jihad-e-Keshavarzi Research Center, Shahrekord, Iran. To prepare the aqueous extract, chamomile plants were collected at the flowering stage from chamomile farms in Isfahan province, Iran (32°38′30″N, 51°39′40″E, and an altitude of about 1570 m above sea level). Plants were randomly selected and taken from a depth of 20–30 cm in the soil using hand shovels. Then the samples were immediately packed in plastic bags and dried in the shade at temperature of 25 °C in the laboratory. After drying the plant organs, they were separately ground to become a powder and softened. Qasem, 2017, Carrubba et al., 2020 method was used to determine the plant extract. Then, to prepare a solution in a ratio of 1:10, 50 g of each of the mentioned plant parts (leaves + stems (shoot), roots, and whole plant) were added separately to 0.5 L of distilled water (for extraction in the laboratory from distilled water as a solvent). The prepared extract was then refrigerated for 48 h in a completely dark environment, then passed through a double-layered cotton cloth and through Whatman filter paper No. 1 to obtain smooth and pure extracts, which were used as extracts. These prepared extracts were considered as extract 100%. The extract was then diluted with distilled water at concentrations of 50, 100, and 150 mL/L. The electrical conductivity of the extracts was measured using an EC meter.

Five mL of each concentration of the extract was added to various different Petri dishes containing both wheat seeds and flixweed. Distilled water was also considered as control (zero concentration), and for similarity with natural conditions, extraction was carried out in cold water (ambient temperature) (Mahmoud et al., 2016, Qasem, 2017).

Petri dishes of 180 mm in diameter were used for each treatment. Two layers of Whatman filter paper No. 2 were placed in the petri dishes. Then, 25 seeds of flixweed were sown in each Petri dish. Wheat sowing was also similar to flixweed. After adding the extracts with different concentrations from each plant organ, the lids were placed on top of the Petri dishes, and they were placed in a growth chamber with dark conditions, at 22 ± 2°C and humidity of 70%. Six d after the beginning of the experiment, all seedlings were considered for the following parameters measurement.

### 2.3 Measurements and calculations

Traits measured included extract compositions, germination rate, germination percentage, hydrogen peroxide (H_2_O_2_), lipid peroxidation, proline content, mitotic index (MI), cell viability, radicle and plumule length, along with seedling length, dry weight, and vigor index.

#### 2.3.1 Liquid chromatography-mass spectrometry analysis

Extract compositions were determined by the Liquid chromatography (LC)-mass spectrometry (MS) method in two phases of positive and negative using a Waters Alliance 2695 HPLC-Micromass Quattro micro API Mass Spectrometer. The process requires both equipment (LC and MS) After inserting a sample, extracts passed through the LC and then identified with the MS. All samples were separated at Atlantis of T3-C18 3µ, 2.1 × 100 mm, 35 °C and flow rate of 0.2 mL/min with injection volume 5 µL, collision energy of 30 eV, flow gas of 200 L/h, and desolvation temperature of 300 °C.

#### 2.3.2 Germination rate

The radicle length of at least 2 mm was considered as the germination phase. The germination rate was calculated using the following formula (Kanjevac et al., 2022):

(1) $$Germination\;rate\;\left(GR\right)\;=\frac{G1}{N1}+\frac{G2}{N2}+\cdots +\frac{\mathrm{Gn}}{\mathrm{Nn}}$$

G_1_, G_2_, and G_n_ are the number of germinated seeds calculated at the first, second and last counts; N_1_, N_2_, and N_n_ are the number of days after sowing to the first, second, and last counts.

#### 2.3.3 Germination percentage

The germination percentage was also calculated according to the following formula:

(2) $$Germination\;percentage\;\left(GP\right)\;=\;100\times \frac{n}{N}$$

In this equation, n is the number of seeds germinated after the sixth d, and N is the total number of seeds sown (Ikic et al., 2012).

#### 2.3.4 Hydrogen peroxide

To determine the amount of hydrogen peroxide (H_2_O_2_) according to method of Nag et al. (2000), one g of seedling tissue was homogenized with 17 mL of cold acetone. Then, a titanium reagent was added to obtain the final concentration (4%). To precipitate the titanium peroxide complex, 0.2 mL of concentrated ammonium was added per one mL of the reaction mixture. The mixture was centrifuged in a refrigerated centrifuge at 8500 rpm for five min. The precipitate was washed twice with five mL of acetone and then dissolved with two mL of one M sulfuric acid. The absorption spectrum of the Ti-H_2_O_2_ complex, which is yellow-orange, was read at 410 nm by a spectrophotometer. The adsorption rate was calculated using the H_2_O_2_ standard curve.

#### 2.3.5 Lipid peroxidation

To estimate the rate of lipid peroxidation of the membrane according to the method by Nag et al. (2000), 250 mg of fresh seedling tissue was ground with five mL of 0.1% trichloroacetic acid in a mortar. The extract was centrifuged at 8500 rpm for 10 min at 4 °C. To one mL of the centrifuged supernatant was added five mL of a 20% solution of trichloroacetic acid with 0.5% of thiobarbituric acid. The resulting mixture was placed in a hot water bath at 95 °C for 30 min and then immediately placed in an ice bucket for 10 min. Then, the samples were centrifuged (10,000 rpm, 4 °C, 15 min). The absorbance intensity of this solution was recorded using a spectrophotometer at 535 nm. The material absorbed at the above wavelength is a red complex. The absorbance of other non-specific pigments was read at 600 nm and subtracted from this value.

#### 2.3.6 Proline content

The proline content of samples was determined using the method described by Bates et al. (1973). About 100 mg of each sample was prepared and added to three mL of sulfosalicylic acid (3% w/v) and then centrifuged (2000 rpm, 10 min). The supernatant (one mL) was mixed with ninhydrin acid and frozen acetic acid in the ratio of 1:1:1 and incubated in boiling water for one h and then in an ice bath for 10 min. Toluene (4 mL) was added to the mixed solution, and the organic and mineral phases were separated. The organic phase was examined at 520 nm by spectrophotometer. The proline content was recorded for the calibration line prepared with pure proline.

#### 2.3.7 Mitotic Index (MI)

After flixweed and wheat seeds were treated with the chamomile extract, radicles were collected and fixed in the carnoy's liquid for 24 h, then hydrolyzed with 5% hydrochloric acid for five min. Finally, the chromosomes were stained with an aceto-orcein reagent for 30 min, and different methods were examined.

Root tips with approximately-two mm were squashed and then visualized by an inverted microscope equipped with a digital camera. A minimum of 1000 cells from each treatment with three replicates was analyzed. The proportion of the number of cells in mitosis to the total cells observed was calculated as the mitotic index (MI). The ratios of cells in mitotic stages were also calculated (Yan et al., 2015).

#### 2.3.8 Cell viability

The cell viability of the root was evaluated with Evans blue (EB) staining. After treatment, the segments (∼1 cm of the root tip) of wheat and flixweed seedlings were cut and stained separately with 0.25% (w/v) EB for one h at 25 ± 2 °C. After washing with double distilled water, the stained root segments were soaked in one mL of N, *N*-dimethylformamide for 24 h at 25 °C in the dark to extract the EB that had been absorbed into the wheat and flixweed root segments. The absorbance of the released EB was measured at 600 nm using a spectrophotometer. The EB absorbance was calculated as the ratio between OD (optical absorbance) of the treated and control groups (Yan et al., 2015).

#### 2.3.9 Seedling vigor index

The seedling vigor index, which is a suitable criterion for estimating seedling strength, was determined using Eq. (3) (Islam et al., 2009):

(3) Seedlingvigorindex(SVI)=(GP×SR)/100

In this equation, GP and SR are germination percentage and seedling length, respectively.

### 2.4 Statistical analysis

For each plant, data were analyzed as a factorial experiment (3 × 3) in a completely randomized design (CRD) with three replicates. To compare wheat with flixweed, all data in each plant were divided into zero concentration (control) and then multiplied by 100 so that the data and mean values were compared as the % control. The analysis was performed using Proc GLM in SAS v.9.4 statistical software. When significant, mean values were compared using the least significant difference (LSD) method at the 5% probability level. It should be noted that for further communication and comparison of the two plants in the experiment, T-test was performed.

## 3 Results

### 3.1 Chamomile bioactive compounds

Liquid chromatography–mass spectrometry (LC/MS) analysis of chamomile extracts showed that these extracts contained a mixture of flavonoids, phenolic compounds, terpenes, and alkaloids, so that most of the compounds present were flavonoids (root 25.9%, shoot 30.0% and root + shoot 27.5%), phenol (root 15.69%, shoot 25% and root + shoot 22.2%) and terpenes (root 15.7%, shoot 18.5% and root + shoot 17.1%) (Table S1). In addition, 3.7, 6.7, and 3.9% of the root, shoot, and root + shoot extracts were alkaloids, respectively. Also, the chamomile extract contained 3.3, 3.7, and 1.9% saponin in the root, shoot, and root + shoot, respectively. Laminin, quinone, and coumarin were also found in shoot extracts. Coumarins which possess several biological properties such as antimicrobial, antioxidant, antiinflamitory, and enzyme inhibitory activity (Malikov et al., 1998), were also found throughout the plant (Table S1). Other compounds observed included some unknown compounds from the flavonoid group, 3-methyl cyclohexanol, malic acid, *N*-nonanol, neryl acetate, or bornyl acetate, nerolidol, P-cymene, cycloartenol, bisabolone oxide, limonene-6-ol, pivalate, hexadecanoic acid, 4-vinylpheno, patuletin, dihydroxy-tetramethoxy flavone, amitrole (amino-1,2,4-triazole-3). The amount of these components was higher in the shoot than in the root chamomile (Table S1).

### 3.2 Germination rate

**Fig. 1:**
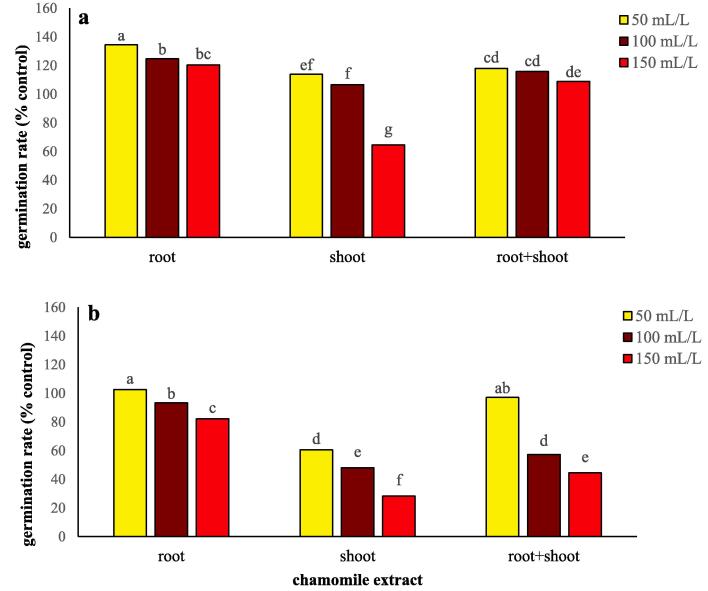
Changes in germination rate of wheat (a) and flixweed (b) treated with different types and concentration of chamomile extract. Different letters above the bars indicate significant difference at *P* < 0.05.

Chamomile extract stimulated the germination rate of wheat and flixweed seeds (Table 1). Extract type, concentration, and extract type × concentration significantly (*P* ≤ 0.01) affected the germination rate of flixweed and wheat (Table 1). Therefore, the interaction of extract type × concentration is discussed. An increase in the concentration of aqueous extract from 50 to 150 mL/L decreased flixweed germination regardless of the extract type (Fig. 1b). Addition of 150 mL/L of chamomile shoot resulted in the lowest germination rate of flixweed (72.0% decrease compared to the control) (Fig. 1b). Our data indicated that root and root + shoot at 50 mL/L concentrations were the least effective in reducing flixweed germination rate. When shoot was used, 50% less concentration was required to decrease flixweed germination rate indicating the aboveground section of chamomile contributes more to controlling weeds than the belowground.

**Table 1:** Stimulation and inhibition of germination rate, germination percentage, hydrogen peroxide, malondialdehyde and proline content (% of control) of wheat and flixweed in the presence of different types and concentrations of chamomile extract.

	Germination percentage(% of control)		Germination rate(% of control)		Hydrogen peroxide(% of control)		Malondialdehyde (% of control)		Proline content(% of control)	
	Wheat	Flixweed	Wheat	Flixweed	Wheat	Flixweed	Wheat	Flixweed	Wheat	Flixweed
*Extract type*										
Root	98.70^a^	99.14^a^	126.5^a^	92.69^a^	120.4^c^	107.6^b^	116.9^c^	184.0^c^	163.4^c^	340.9^b^
Shoot	89.07^b^	81.57^b^	95.04^C^	45.63^c^	210.7^a^	159.4^a^	176.9^a^	573.2^a^	758.4^a^	1522^a^
Root + shoot	94.26^a^	97.05^a^	114.2^b^	66.30^b^	149.0^b^	116.7^b^	141.9^b^	376.5^b^	314.7^b^	499.1^b^

*Extract concentration (mL/L)*										
50	106.7^a^	101.4^a^	122.1^a^	86.75^a^	146.0^c^	117.7^c^	135.1^b^	317.3^c^	345.4^c^	376.8^c^
100	92.78^b^	96.42^b^	115.7^b^	66.20^b^	157.8^b^	124.9^b^	141.1^b^	376.9^b^	412.5^b^	609.2^b^
150	82.59^c^	79.93^c^	98.00^c^	51.66^c^	176.4^a^	141.1^a^	159.5^a^	439.5^a^	478.5^a^	1376^a^

*ANOVA*										
Extract type (E)	**	**	**	**	**	**	**	**	**	**
Extract concentration (C)	**	**	**	**	**	**	**	**	**	**
E × C	NS	**	**	**	*	**	NS	NS	**	**

In each group, means with similar letters were not statistically significant at *P* < 0.05 (LSD test). NS, ** and * non-significant and significant at *P* < 0.01 and at *P* < 0.01, respectively.

Similarly, the wheat germination rate was the lowest (35.4% lower than the control) at the 150 mL/L shoot extract (Fig. 1a). This indicates that a high concentration of aqueous extract from shoot could decrease wheat production while decreasing flixweed germination. Based on our data, the application of 150 mL/L of root + shoot extract could significantly reduce flixweed germination while stimulating wheat germination (Fig. 1a).

### 3.3 Germination percentage

**Fig. 2:**
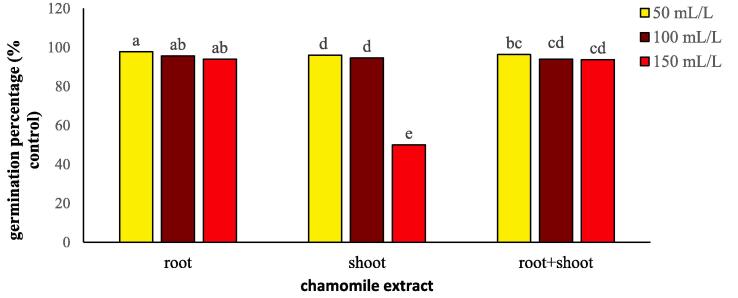
Changes in germination percentage of flixweed treated with different types and concentration of chamomile extract. Different letters above the bars indicate significant difference at *P* < 0.05.

Germination percentage of flixweed was significantly (*P* ≤ 0.01) influenced by extract type, concentration, and extract type × concentration of chamomile (Table 1). Among all extract types, roots had the most negligible effect on flixweed germination (Fig. 2). Chamomile shoot at 150 mL/L decreased flixweed germination higher than any other extract type and rate reflecting on allelopathic compounds in the shoot (Fig. 2). An increase in root and root + shoot extract concentration only slightly decreased flixweed germination indicating for effective control of flixweed, shoot extract is required. However, our data indicate that wheat germination could also be affected by the high concentration of extracts regardless of the extract type (Table 1).

### 3.4 Hydrogen peroxide

**Fig. 3:**
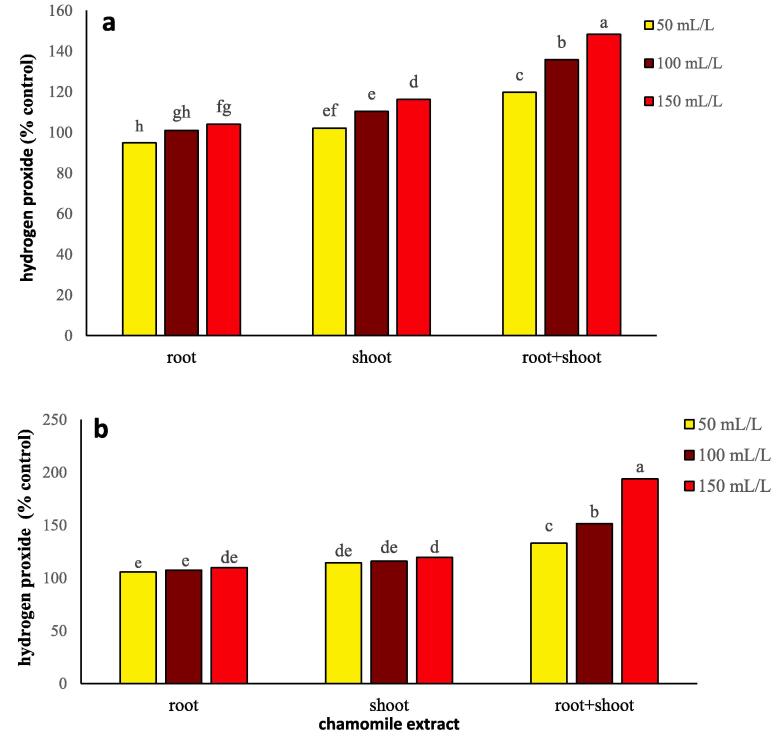
Changes in hydrogen peroxide production of wheat (a) and flixweed (b) treated with different types and concentration of chamomile extract. Different letters above the bars indicate significant difference at *P* < 0.05.

Chamomile extract type and concentration effects on the hydrogen peroxide content in wheat and flixweed were significant (*P ≤* 0.01) and their interaction in flixweed (*P ≤* 0.01), and wheat (*P ≤* 0.05) was significant (Table 1). The highest hydrogen peroxide content was observed in root + shoot extracts with 150 mL/L in wheat (48%) and flixweed (94%) compared to the control. The T-test results indicated that the amount of hydrogen peroxide in flixweed after applying chamomile root + shoot extracts was much higher than that of wheat. (Fig. 3a, b; Table S2).

### 3.5 Lipid peroxidation

Malondialdehyde (MDA) concentration indicates lipid peroxidation of the membrane. According to Table 1, the effects of chamomile extract type and concentration on malondialdehyde concentration in wheat and flixweed were significant (*P ≤* 0.01), but the extract type × concentration interaction for both plants was not significant (Table 1). MDA concentration was highest in flixweed and wheat when chamomile extract from the shoot was used (Table 1). T-test results indicated that the concentration of MDA in wheat was much lower than that of flixweed. MDA concentration ranged from 135.1% (at 50 mL/L chamomile extract concentration) to 159.5% (at 150 mL/L chamomile extract concentration) in wheat. An increase in chamomile extract concentration from 50 to 150 mL/L, increased MDA concentration in flixweed from 317.3 to 439.5% (Tables 1 and S2). Regardless of the plant parts, the amount of MDA in flixweed was three times higher than in wheat (Table 1).

### 3.6 Proline content

**Fig. 4:**
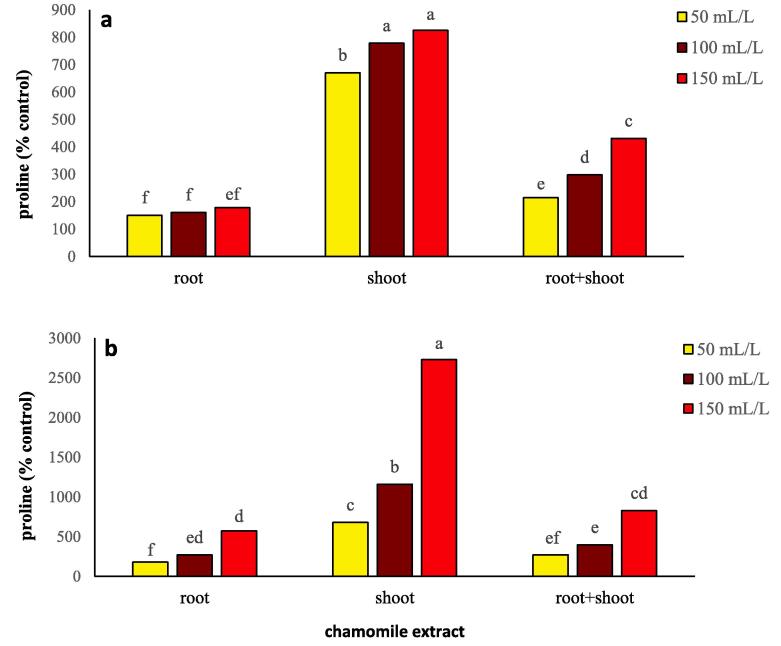
Changes in proline content of wheat (a) and flixweed (b) treated with different types and concentration of chamomile extract. Different letters above the bars indicate significant difference at *P* < 0.05.

Extract type, concentration, and extract type × concentration significantly (*P ≤* 0.01) affected the proline content of flixweed and wheat (Table 1). In flixweed, application of chamomile extracts at 150 mL/L consistently increased proline content compared to the lower concentrations, with the highest proline content recorded from shoot extracts at 150 mL/L concentration (Fig. 4b). Only in shoot extracts an increase in proline content was observed with an increase in concentration from 50 to 100 mL/L (Fig. 4b). In wheat, the highest accumulation of proline was recorded from the addition of shoot extracts at 100 and 150 mL/L concentrations. The lowest accumulation of proline content was recorded from root extracts regardless of the concentration (Fig. 4a; Table S2).

The production of proline in flixweed by application of shoot (184%) and root extracts (124%), was higher than that of wheat (Tables 1 and S2). At a concentration of 150 mL/L of chamomile extract, the proline content of flixweed seedling was three times that of wheat seedling (Fig. 4a, b; Table S2).

### 3.7 Mitotic index

**Fig. 5:**
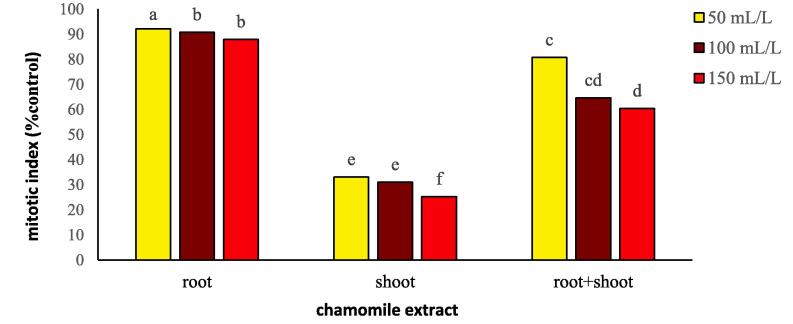
Changes in mitotic index of wheat treated with different types and concentration of chamomile extract. Different letters above the bars indicate significant difference at *P* < 0.05.

In this experiment, various and even modified methods were used to measure the mitotic index of flixweed, but due to the small size of flixweed cells and lack of proper resolution, only the results of the wheat mitotic index were recorded. Wheat seedling mitotic index was significantly affected by the type and concentration of aqueous extract of chamomile and the interactions of these two factors (*P ≤* 0.01) (Table 2). As shown in Fig. 5, at a concentration of 50 mL/L of root extract, the measured mitotic index of wheat root tip was more than 92%. Application of other concentrations of other extracts also reduced this index in wheat. At the highest concentration (150 mL/L) of chamomile shoot, the mitotic index was decreased by 76.7% compared to the 50 mL/L of root extract (Fig. 5).

**Table 2:** Inhibition of mitotic index (MI), prophase index (PI), metaphase index (MeI), anaphase index (AI) and telophase index (TI) (% of control) of wheat in the presence of different types and concentrations of chamomile extract.

	MI (% of control)	PI (% of control)	MeI (% of control)	AI (% of control)	TI (% of control)
*Extract type*					
Root	68.57^a^	95.49^a^	87.37^a^	85.01^a^	86.13^a^
Shoot	29.80^c^	78.96^c^	66.35^b^	65.98^c^	51.31^c^
Root + shoot	42.11^b^	90.29^b^	75.48^b^	72.33^b^	65.77^b^

*Extract concentration (mL/L)*					
50	53.05^a^	90.21^a^	82.06^a^	77.83^a^	71.35^a^
100	46.04^b^	88.67^b^	77.31^a^	73.59^ab^	67.15^a^
150	41.38^c^	85.86^c^	69.83^b^	71.90^b^	64.72^a^

*ANOVA*					
Extract type (E)	**	**	**	**	**
Extract concentration (C)	**	**	**	*	NS
E × C	**	NS	NS	NS	NS

In each group, means with similar letters were not statistically significant at *P* < 0.05 (LSD test).

NS, and ** are non-significant and significant at *P* < 0.01, respectively.

The process of wheat cell division in response to the type of extract was similar to the mitotic index (Table 2). In response to the concentration of the extract, only the prophase process was similar to the mitotic index. In other stages of cell division, no significant difference was observed between the 50 and 100 mL/L concentration. Except the telophase stage, which did not show a significant response to the extract concentration, in other stages of cell division, the lowest was obtained with the highest concentration (Table 2).

### 3.8 Cell viability

**Fig. 6:**
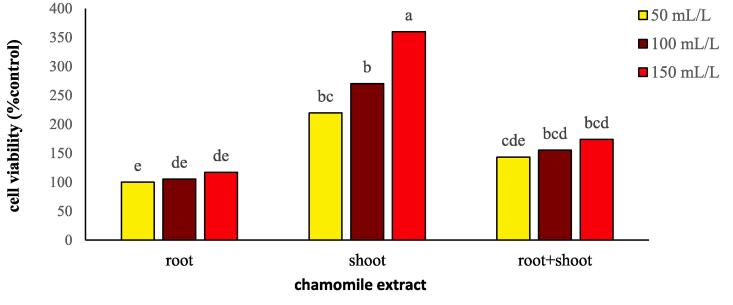
Changes in cell viability of wheat treated with different types and concentration of chamomile extract. Different letters above the bars indicate significant difference at *P* < 0.05.

Evans blue (EB) uptake indicates cell viability. The main effects of type and concentration of chamomile extract on wheat and flixweed cell viability were significant (*P ≤* 0.01), but the interaction of type × concentration of chamomile extract was only significant (*P ≤* 0.01) in wheat (Table 3). A comparison of the absorption rate of EB in two plants showed that in type of extract and each at each concentration, the absorption rate in flixweed was much higher than that of wheat (Tables 3 and S2). T-test results indicated that by increasing the concentration of chamomile extract from 50 to 150 mL/L, a significant difference was observed in the absorption of EB in flixweed compared to wheat, so that at the maximum concentration in wheat by 54% reduction in adsorption compared to flixweed was recorded. Also, the use of shoot extract reduced flixweed by 56% compared to wheat (Tables 3 and S2). With the application of 150 mL/L of shoot extract, the absorption rate of EB was about three times higher than the use of root extract at the same concentration (Fig. 6).

**Table 3:** Stimulation and inhibition of relative Evans blue uptake, radicle length and plumule length (% of control) of wheat and flixweed in the presence of different types and concentrations of chamomile extract.

	Relative Evans blue uptake (% of control)		Radicle length (% of control)		Plumule length (% of control)	
	Wheat	Flixweed	Wheat	Flixweed	Wheat	Flixweed
*Extract type*						
Root	174.3^c^	215.4^c^	103.8^a^	76.91^a^	88.37^a^	79.33^a^
Shoot	408.01^a^	653.2^a^	82.97^b^	41.90^c^	66.09^c^	54.43^c^
Root + shoot	252.1^b^	368.5^b^	83.05^b^	50.50^b^	81.73^b^	70.09^b^

*Extract concentration (mL/L)*						
50	229.3^b^	359.3^c^	103.6^a^	65.75^a^	90.33^a^	72.46^a^
100	259.3^b^	400.4^b^	90.73^b^	57.72^b^	79.12^b^	68.91^b^
150	345.8^a^	477.5^a^	75.48^c^	45.83^c^	66.74^c^	62.47^c^

*ANOVA*						
Extract type (E)	**	**	**	**	**	**
Extract concentration (C)	**	**	**	**	**	**
E × C	**	NS	NS	NS	NS	NS

In each group, means with similar letters were not statistically significant at *P* < 0.05 (LSD test). NS, ** and * non-significant and significant at *P* < 0.01 and at *P* < 0.05, respectively.

### 3.9 Radicle length

Extract type, concentration, but not extract type × concentration significantly (*P ≤* 0.01) affected the radicle length of flixweed and wheat (Table 3). The extracts of different parts of chamomile reduced radicle length compared to the control (Table 3). Among chamomile plant parts, shoot extract resulted in the greatest reduction of radicle length in both wheat and flixweed, but the effects in flixweed were more severe than that of wheat (Table 3). Chamomile extract concentration had an inhibitory effect on the radicle growth of wheat and flixweed (Table 3). An increase in the extract concentration of chamomile to 150 mL/L, decreased radicle growth of flixweed and wheat by 54 and 29% compared to the control (Table 3).

### 3.10 Plumule length

The plumule length was affected by extract type and concentration (*P ≤* 0.01(, but not the interaction of extract type × concentration (Table 3). The extracts of different parts of chamomile reduced plumule length compared to the control and the effect of shoot extract was more than other extracts and in flixweed was more severe than in wheat.

The highest plumule length of wheat was observed in 50 mL/L concentration and chamomile root extract. In addition, the maximum concentration extract reduced the plumule length of the wheat by 26% (Table 3). Exposure of flixweed seeds to chamomile extract reduces its plumule length by 20–45%. Furthermore, at the minimum and maximum concentrations of chamomile extract, the plumule length can be reduced by 27–38%, respectively (Table 3).

### 3.11 Seedling length

Extract type, concentration, but not extract type × concentration significantly (*P ≤* 0.01) affected the seedling length of flixweed and wheat (Table 4). Our data indicated that chamomile shoot extract reduced wheat seedling length by 30 and flixweed seedling length by 52%. Even chamomile root extract resulted in a 22% reduction in flixweed seedling length (Table 4). The addition of 150 mL/L concentration decreased the seedling length of wheat and flixweed by 28 and 49%, respectively, compared to the control (Table 4).

**Table 4:** Stimulation and inhibition of seedling length, seedling dry weight, seedling vigor index (% of control) of wheat and flixweed in the presence of different types and concentrations of b chamomile extract.

	Seedling length (% of control)		Seedling dry weight (% of control)		Seedling vigor index (% of control)	
	Wheat	Flixweed	Wheat	Flixweed	Wheat	Flixweed
*Extract type*						
Root	89.62^a^	77.83^a^	82.45^a^	74.58^a^	90.23^a^	77.35^a^
Shoot	67.48^b^	47.66^c^	49.39^b^	36.25^c^	61.05^b^	40.55^c^
Root + shoot	88.07^a^	52.32^b^	68.80^b^	54.97^b^	83.85^a^	51.06^b^

*Extract concentration (mL/L)*						
50	94.32^a^	69.15^a^	74.22^a^	60.17^a^	101.31^a^	70.30^a^
100	82.77^b^	58.12^b^	66.80^b^	54.49^b^	77.07^b^	56.29^b^
150	68.09^c^	50.55^c^	59.62^c^	51.14^c^	56.74^c^	42.37^c^

*ANOVA*						
Extract type (E)	**	**	**	**	**	**
Extract concentration (C)	**	**	**	**	**	**
E × C	NS	NS	**	NS	NS	**

In each group, means with similar letters were not statistically significant at *P* < 0.05 (LSD test). NS, ** and * non-significant and significant at *P* < 0.01 and at *P* < 0.05, respectively.

### 3.12 Seedling dry weight

The seedling weight of flixweed and wheat showed a significant response to the type and concentration of chamomile extract (Table 4). The greatest dry weight reduction (64% reduction compared to control) was related to flixweed under the shoot extract. In addition, at different concentrations of the chamomile extract, a 40 to 49% flixweed seedling dry weight decrease was observed compared to the control (Table 4).

**Fig. 7:**
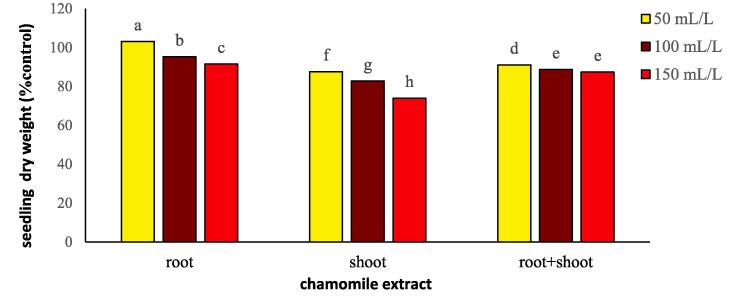
Changes in seedling dry weight of wheat treated with different types and concentration of chamomile extract. Different letters above the bars indicate significant difference at *P* < 0.05.

The interaction between the type and concentration of chamomile extract in wheat showed that adding a 50 mL/L of root extract, increased wheat seedling weight by 3% compared to the control. The lowest seedling weight (26% reduction compared to the control) was related to the concentration of 150 mL/L of chamomile shoot extract (Fig. 7).

### 3.13 Seedling vigor index

**Fig. 8:**
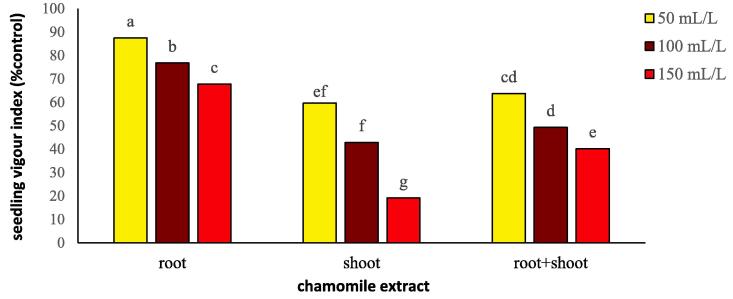
Changes in seedling vigor index of flixweed treated with different types and concentration of chamomile extract. Different letters above the bars indicate significant difference at *P* < 0.05.

Extract type, concentration, and extract type × concentration significantly (*P ≤* 0.01) affected the seedling vigor index (Table 4). Regardless of extract type, seedling vigor index was the lowest at the maximum extract concentration, indicating the inhibitory effect of chamomile extracts on germination percentage and seedling length of flixweed (Table 4; Fig. 8). Among all extract types, the shoot had the strongest inhibitory effect on seedling vigor, especially at the highest chamomile concentration (150 mL/L) (Fig. 8).

The interaction effect of plant type and concentration on wheat was not significant (Table 4), but the mean comparison of extract type on wheat showed that root and root + shoot extracts had the least adverse effects compared to the shoot. The inhibitory effect of 150 mL/L concentration was greater by 28% than that of 50 mL/L concentration.

## 4 Discussion

The absence of visible changes in the EC of the extracts assessed in this study compared to distilled water indicated that the inhibitory effects of aqueous chamomile extract are independent of EC. Therefore, changes in the cellular processes of flixweed and wheat seedlings due to the phytotoxic impacts are not related to the salts of the extracts (Caser et al., 2020).

The presence of pantothenic acid (vitamin B5) in the root extract and to a lower extent in the whole chamomile extract (Table S1) can show some stress tolerance in seedlings treated with root extract, because vitamin Bs are water-soluble and can play a significant role in inhibiting ROS and indirect stimulation of proline production, which are involved in plant exposure to stress in the phosphate pentose pathway (Burguieres et al., 2007, Fardet et al., 2008).

The presence of Magnoflorine compounds (in shoot extract and to a lower extent in roots), Myristicin (in shoot extract), and Norargemonine (whole chamomile plant) (Table S1) indicates the role of alkaloid inhibitors on the percentage and germination index of flixweed, and therefore reduced the rate of these indices and affected seedling growth. Other studies have shown that allelopathy affects respiration, alters membrane permeability, and inhibits cell growth and enzyme activity (Azirak and Karaman, 2008, Putman et al., 1983). Zeng et al. (2008) introduced toxic gases, organic acids, aromatic acids, aldehydes, simple unsaturated lactones, alkaloids, coumarins, tannins, terpenoids, flavonoids, and steroids as allelopathic compounds. In this regard, phenolic compounds and flavonoids are the main allelochemicals of the plant in the ecosystem and play a significant role in allelopathy (Jacob and Sarada, 2012). They affect cell membrane permeability, inhibit cell division, and interfere with several enzyme activities and major physiological processes, such as protein synthesis and metabolism of some other secondary metabolites (Kato-Noguchi and Kurniadie, 2022).

Experimental data strongly suggest that phenols have several physiological effects that lead to general cytotoxicity, often with nonspecific permeability changes in the cell wall membrane. The ring structure of flavonoids easily forms a stable free radical that can easily cause dimers formation, oligomers, and concentrated tannins (Guevara et al., 2018). The natural functions of terpenoids, monoterpenes, and sesquiterpene are very diverse, with potent inhibitory effects on plant growth and seed germination. In this study, terpene alcohols such as cycloartenol and spathulenol were found in root and root + shoot of chamomile; bisabolol and limonene were present in shoot, and terpene acetates were found in the shoot and whole chamomile plant. Melilotigenin, chamazulene, alpha-amorphene, farnesol, and P-cymene were also present in the shoot of chamomile. At the same time, hederagenin glycoside, camphene, α-terpinene, γ-terpinene, myrcene, and 1,8-cineole were found in roots and shoot of chamomile (Table S1). Biologically active phenolic coumarins (herniarin and umbelliferone) (Table S1) significantly inhibit root growth, and decrease cell activity and the number of cell bodies. We observed these effects in flixweed and wheat seedlings that were treated with extracts driven from the shoot of chamomile (Table 2, Table 3).

Decreased germination rate can be attributed to the role of volatile terpenes in allelopathic features (Beres and Kazinczi, 2000). Volatile terpenes can prevent or delay cell division (Table 2). Other compounds in chamomile extracts, found in our study, such as 1,8-cineole, limonene, camphene, camphor and P-cymene (Table S1), have different effects such as swelling of the radicle tip, inhibition of plant respiration, and formation of mitochondrial DNA, cessation of mitosis and inhibition of synthesis.

With increasing the extract concentration, the seed germination rate in both species significantly decreased, but this reduction was greater in flixweed (Table 1). Probably due to the inhibitory effect of the allelochemicals as mentioned earlier in chamomile (polyphenols, coumarins, terpenoids, and alkaloids) (Table S1) on gibberellin (Nakabayashi et al., 2022), the flixweed and wheat growth was disrupted by interfering of critical physiological processes such as change of cell structure, change of membrane permeability, inhibition of cell division (Table 2) and the activity of some enzymes and the balance of plant hormones in both radicle and plumule. Different concentrations of aqueous extract of chamomile increased the time required for germination by disturbing the hormonal balance of seeds, affecting cell division and radicle growth (Table 2, Table 3; Fig. 5). The process of germination has different stages and the inhibitory effect of the extract concentration can be attributed to the reduction of the embryo use of reserve compounds, germination power and seedling growth (Yamamoto et al., 1997). Tomaszeweski and Thimann (1996) also state that many alleloochemicals reduce the stimulatory effect of endoplasmic growth indole-3-acetic acid and gibberellin, which reduces the growth of plant organs. Various mechanisms of uptake from roots and surface cells and different metabolic pathways and sites of action may explain the differences in plant susceptibility to the same allelochemicals.

Seedling growth depends partly on the transfer of storage compounds from the seed. During the germination stage, the mobility of reserve compounds affected by allopathic compounds is likely to stop or be delayed, so the growth rate and the seedling weight of wheat and flixweed decreases (Table 4). Wei et al. (2019) reported the volatile oil released by Atriplex cana significantly inhibited the growth of seedlings of four weed species, including *Amaranthus retroflexus* (L.) and *Poa annua* (L.), and it had a high value for further use as a biological herbicide. In plants, several types of ROS play an essential role in controlling processes such as growth, response to environmental and abiotic stimulators, stomatal behavior, and pathogen defense. However, under stress conditions, overproduction of ROS can lead to oxidative damage to proteins, lipids, and DNA. Also, natural chemical products can increase ROS production in the receptor plants (Apel and Hirt, 2004). The addition of chamomile extracts produced ROS in wheat and flixweed seedlings (Table 1), which may have interfered with the electron transfer chain. In our study, the addition of shoot extracts from chamomile resulted in a higher amount of hydrogen peroxide in flixweed than in wheat, reflecting in a higher lipid peroxidation in flixweed at higher concentrations of shoot extract (Table 1). Excessive ROS can cause cell membrane dysfunction, damage to cell viability, and increased peroxidation of cellular lipids, followed by decreased cell vitality and cessation of mitotic stages in these plants, especially flixweed (Table 2). On the other hand, ROS probably played a significant role in inhibiting growth, which could be related to the presence of flavonoids in chamomile extract such as quercetin, apigenin, patuletin, luteolin, coumarin, kampferol, rutin, taxifolin (Table S1).

However, the literature suggests that oxidative damage alone cannot be responsible for the phytotoxic effects of other plants. An increase of ROS, alteration of membrane permeability and cell structure, change of photosynthesis and respiration, and inhibition and/or reduction of seed germination and seedling growth, are documented as effects of plant extract (Radhakrishnan et al., 2018). Therefore, the impact of allelopathy is indicative of a change in a large set of metabolic processes (Cruz-Ortega et al., 2007). Yogatek et al. (2006) demonstrated that extract of sunflower (*Helianthus annuus*) destroyed cell membranes and reduced activity of antioxidant enzymes of wild mustard (*Sinapis arvensis*) seedling.

The increase in MDA concentration is a direct result of the abundance of lipid peroxidation, including membrane lipids (Fu et al., 2022). The degradation of these lipids reduces the cohesion and stability of cytoplasmic membranes. Therefore, chamomile extract by affecting membrane lipid peroxidation and oxidative damage (Table 1; Fig. 3a, b) and, as a result of breaking down fats, changes the fluidity and permeability of membrane lipid layers and can dramatically change cell integrity (Table 1).

Proline acts as a storage reservoir of nitrogen or solute that reduces the osmotic potential of the cytoplasm and helps the plant to tolerate stress (Gajewska et al., 2006). In wheat and flixweed, free proline accumulates in response to allelochemical stress. It can act as a molecule's signaling to modulate several functions such as osmotic regulation, detoxification of ROS, and preservation of membrane integrity. Proline accumulation also is associated with the presence of ROS, which in the present experiment, hydrogen peroxide (Fig. 3a, b) induced by chamomile extract produced more proline in flixweed than wheat, and the application of higher concentrations of shoot extract reduced these oxidants (Fig. 4a, b).

In addition, more phenols were observed in chamomile shoot extract compared to roots (Table S1); therefore, the greater inhibitory role of phenolic compounds in preventing germination and radicle elongation can be confirmed, and consequently disrupts the normal growth of both plants, especially flixweed. Organic acids in chamomile extract such as citric acid, malic acid, quinic acid, hexadecanoic acid, vanillic acid, gallic acid, ferulic-glucose (in shoot extract) or m-coumaric acid, indoleacetic acid, p-coumaric acid, octadecanoic acid, ferulic acid, chlorogenic acid, and caffeic acid (in root + shoot extract), which are subsets of phenolics (Table S1) prevent the germination of many seeds and create the allelopathy effects on various physiological processes in plant, incuding inhibition of cell division, elongation and microscopic structures, inhibition of endogenous hormone synthesis and protein synthesis.

Some chemical agents can prevent mitosis and affect DNA synthesis, cyclin-dependent kinase activity, and microtubules (Planchais et al., 2000). Since the number and amount of compounds such as polyphenols, flavonoids, coumarins, terpenoids, quinones, and alkaloids (Table S1) were higher in shoot extract than in other extracts, shoot extract is likely to affect vital enzymatic functions, protein synthesis, respiration transcription, membrane stability, electron transfer, signal transduction, synthesis of endogenous plant hormones, inhibition of cell division and replication, and finally by binding to the processing enzymes of DNA or DNA/RNA, can be more than extracts disrupt replication or transcription (Table 2; Fig. 5).

The rapid decline of the cells number in all mitotic stages in wheat root indicated that these extract compounds might interfere with a specific phase of the cell division cycle and inhibit cells from entering mitosis. Thus, inhibiting mitosis by this compound is one of the factors that reduce seedling growth (Table 2). In this regard, Owens (1973) showed that coumarin and scopoletin, reduce mitosis in *Poa trivialis*. Flavonoids and coumarin in chamomile extract (Table S1), by inhibiting cell division and elongation of cells in the germination process, inhibit germination and reduce the radicle and plumule length of seeds of flixweed. Therefore, the presence of allopathic compounds such as bioactive phenolic coumarins (herniarin and umbelliferone), flavones (apigenin and apigenin-7-O-glucoside), flavonols (quercetin (quercetin-7-O-glucoside), Isoquercitrin, quercetin hexoside, quercetin-3-o-rhamnoside, quercetin-3-O-glucuronide-7-O-galactoside, quercetin galactoside) and rutin), quinone (1,2-benzoquinone) and other phenolic compounds, flavonoids and tri-carboxylic acids such as malic acid (Table S1) in aqueous chamomile extract by reducing cell membranes and negatively affecting the activity of plant enzymes in the germination stage, reduce the seedling growth of wheat and flixweed.

Therefore, reduced seedling growth in the presence of allopathic compounds is related to severe cessation of mitosis of meristem cells; as a result, it reduces the seedling length (Bertin et al., 2003). Decreased radicle cell viability at high concentrations of shoot extract and double death of flixweed cells compared to wheat indicates the presence of cell division inhibitors in chamomile extract and the different sensitivity of species to these inhibitors (Table 2, Table 3). Low proline levels and presnece of pantothenic acid (vitamin B5) could explain the low inhibitory effects of root extracts as reported in the literature (Berlin et al., 1993).

Overall, our data suggest that chamomile extracts could decrease the population of flixweed by reducing its germination and weakening its seedlings. Suppressing flixweed decreases its competition with wheat resulting in greater wheat growth and grain yields.

## 5 Conclusion

Our data indicated that chamomile extracts could decrease the growth of flixweed. Chamomile’s bioactves disrupts weed growth by increasing the level of oxidants and osmolytes and reducing cell division and viability. This suggests that chamomile can be applied to produce natural herbicides to suppress flixweed in wheat cropping systems. Our research also indicates that chamomile could be planted as a preceding crop in rotation with wheat to reduce flixweed competition with wheat. However, more studies with other crop and weed species are needed to produce this natural herbicide that can be more active or selective in cropping systems.

## Declaration of Competing Interest

The authors declare that they have no known competing financial interests or personal relationships that could have appeared to influence the work reported in this paper.
