# Supplementary material for: Exploring the use of chamomile (Matricaria chamomilla L.) bioactive compounds to control flixweed (Descurainia sophia L.) in bread wheat (Triticum aestivum L.): Implication for reducing chemical herbicide pollution
Source: Saudi J Biol Sci. 2022 Aug 27;29(11):103421. doi: 10.1016/j.sjbs.2022.103421 (PMC9579404; doi:10.1016/j.sjbs.2022.103421)
Supplement: Supplementary data 1 [file mmc1.docx]

Table S1: LC.MS analysis for extract different organs of chamomile.

| **Whole plant** | | | **Aerial parts** | | | **Root** | | |  | | | | | |
| --- | --- | --- | --- | --- | --- | --- | --- | --- | --- | --- | --- | --- | --- | --- |
| **Rt (min)** | **Area %** | **Existence** | **Rt (min)** | **Area %** | **Existence** | **Rt (min)** | **Area %** | **Existence** | **MW** | **m.z [M-H]^+^** | **m.z [M- H]^-^** | **Type** | **Formula** | **Probable Compounds** |
| 3.063 | 0.74 | * | 19.846 | 1.95 | * | 19.294 | 3.04 | * | - |  | 62 | Flavenone | - | Not identified |
| 1.108 | 2.61 | * | 17.703 | 11.55 | * | 19.294 | 1.23 | * | 84.08 |  | 83 | Amine | (C2H4N4) | Amitrole (Amino-1,2,4-triazole-3) |
| 12.433 | 7.62 | * | 19.848 | 12.11 | * | 19.294 | 10.87 | * | - |  | 91 | Flavenone | - | Not identified |
|  |  |  |  |  |  | 3.83 | 8.70 | * | - |  | 105 | - | - | Not identified |
| 20.936 | 3.20 | * |  |  |  | 19.656 | 3.91 | * | - |  | 107 | - | - | Not identified |
| 13.561 | 2.21 | * | 19.846 | 3.91 | * | 10.79 | 1.30 | * | 114.19 |  | 113 | Cyclohexanol derivatives | (C7H14O) | 3‑methylcyclohexanol |
| 3.063 | 5.90 | * | 3.083 | 4.35 | * |  |  |  | 134.8 134.22 |  | 133 | (Phenolic compounds) (Triterpenoid) | (C4H6O5)  ( [C10H14](https://pubchem.ncbi.nlm.nih.gov/#query=C10H14)) | malic acid or Para-Cymene |
|  |  |  |  |  |  | 3.083 | 5.22 | * | - |  | 182 | - | - | Not identified |
|  |  |  |  |  |  | 3.083 | 6.09 | * | 218.3 |  | 217 | Triterpenoid | (C15H24) | β-Bisabolenal |
| 3.063 | 2.46 | * |  |  |  | 3.083 | 6.52 | * | 219.24 |  | 218 | Amide | (C9H17NO5) | Pantothetic acid (B5 vitamin) |
| 3.063 | 2.46 | * |  |  |  | 3.083 | 3.48 | * | 220.35 |  | 219 | Sesquiterpene | (C15H24O) | Caryophyllene oxide or Spathulenol |
|  |  |  |  |  |  | 13.339 | 7.39 | * | 314 |  | 313 | Flavone | (C18H34O4) | Dihydroxy-octadecenoic acid |
|  |  |  |  |  |  | 15.244 | 10.87 | * | - |  | 331 | Phenolic compounds | - | Not identified |
|  |  |  |  |  |  | 20.886 | 10.43 | * | 508 |  | 507 | Flavonol | (C21H20O11) | Tetrahydroxy-dimethooxy-7-O-glucoside |
| 0.876 | 6.54 | * |  |  |  | 11.384 | 6.21 | * | 60.052 | 60 | 219 | Triterpenoid | (C30H50O) | Cycloartenol |
| 0.876 | 5.45 | * | 0.658 | 8.45 | * | 11.384 | 5.86 | * | 142.24 | 142 |  | - | (C9H18O) | n-Nonanal |
|  |  |  |  |  |  | 11.808 | 10.34 | * | 180.245 | 181 |  | Phenolic compounds | (C14H12) | Stilbene |
|  |  |  |  |  |  | 1.311 | 4.83 | * | 226.27 226.41 | 227 |  | (Alkane hydrocarbon) (Flavonols) | (C16H34) | n-Hexadecane or Not identified |
|  |  |  | 7.636 | 4.23 | * | 16.043 | 5.52 | * | 236 | 236, 237 |  | Terpenoids | ([C15H24O2](https://pubchem.ncbi.nlm.nih.gov/#query=C15H24O2))  (C15H24O2) | Bisabolone oxide or Limonen-6-ol ,pivalate |
|  |  |  | 8.352 | 1.97 | * | 16.043 | 10.34 | * | 276.37 | 278 |  | Phenolic compounds | (C16H32O2) | hexadecanoic acid |
|  |  |  |  |  |  | 16.043 | 2.41 | * | 300 | 300 |  | Flavonols | (C16H12O6) | Kaempferide |
|  |  |  | 8.352 | 2.47 | * | 16.043 | 0.69 | * | 332.262 | 332 |  | Flavonols | (C16H12O8) | Patuletin |

Table S1: (continued) LC.MS analysis for extract different organs of chamomile.

| **Whole plant** | | | **Aerial parts** | | | **Root** | | |  | | | | | |
| --- | --- | --- | --- | --- | --- | --- | --- | --- | --- | --- | --- | --- | --- | --- |
| **Rt (min)** | **Area %** | **Existence** | **Rt (min)** | **Area %** | **Existence** | **Rt (min)** | **Area %** | **Existence** | **MW** | **m.z [M-H]^+^** | **m.z [M- H]^-^** | **Type** | **Formula** | **Probable Compounds** |
| 10.871 | 4.55 | * |  |  |  | 11.384 | 3.45 | * | 343 | 343 |  | Alkaloid | (C20H24NO4) | Magnoflorine |
|  |  |  |  |  |  | 11.384 | 3.10 | * | 356.5 | 357 |  | Phenolic compounds | (C21H40O4) | α-Monoolein |
|  |  |  |  |  |  | 16.043 | 3.79 | * | 392 | 392 |  | - | - | Not identified |
|  |  |  |  |  |  | 16.043 | 1.38 | * | 414.7 | 415 |  | Phenolic compounds | (C29H50O) | Phytosterol |
|  |  |  |  |  |  | 16.043 | 4.83 | * | 542 | 542 |  | saponine | (C24H32N404) | Saponin-A (Flavonoid) |
|  |  |  | 4.829 | 1.95 | * |  |  |  | 90.12 |  | 89 | Alcohol | (C4H10O2) | 1,3-Butandiol |
|  |  |  | 1.583 | 4.69 | * |  |  |  | 94 |  | 93 | Phenolic compounds | - | Not identified |
|  |  |  | 1.583 | 7.42 | * |  |  |  | 110.15 |  | 109 | Phenolic compounds | - | Not identified |
|  |  |  | 1.583 | 2.73 | * |  |  |  | 130.18 130.23 |  | 129 | Phenolic compounds | (C7H14O2) (C8H18O) | Propyl butyrate or n-Octanol |
|  |  |  | 1.583 | 1.95 | * |  |  |  | 168.147 |  | 167 | Phenolic compounds | (C8H8O4) | Vanillic acid |
|  |  |  | 1.583 | 1.95 | * |  |  |  | 170 |  | 169 | Phenolic compounds | (C7H6O5) | Gallic acid |
|  |  |  | 1.583 | 1.95 | * |  |  |  | 186.29 |  | 185 | Phenolic compounds | (C11H22O2) (C11H22O2) (C10H19O2) | n-Hexyl-2-methyl butyrate or Hexyl-3-methyl butanoate or Decanoate |
|  |  |  | 1.583 | 1.95 | * |  |  |  | 192.124  192.17 |  | 191 | Phenolic compounds | (C6H8O7) (C7H12O6) | Citric acid or Quinic acid |
|  |  |  | 1.583 | 12.11 | * |  |  |  | - |  | 216 | - | - | Not identified |
|  |  |  | 1.583 | 1.95 | * |  |  |  | 243.2 |  | 243 | Saponin | - | Not identified |
|  |  |  | 1.583 | 1.56 | * |  |  |  | - |  | 277 | - | - | Not identified |
|  |  |  | 9.365 | 3.91 | * |  |  |  | 316 |  | 315 | (Flavonoid)  (Phenolic compounds) | (C16H12O7) (C18H36O4) | Izorhamnetin or Dihydroxy-octadecanoic acid |
|  |  |  | 1.583 | 11.72 | * |  |  |  | 343 |  | 342 | Alkaloid | (C20H24NO4) | Magnoflorine |
|  |  |  | 7.485 | 3.91 | * |  |  |  | 356 356.32 |  | 355 | Phenolic compounds |  | Not identified or Ferulic acid glucose or Cis.trans ferulic acid hexoside or Ferulic acid glucoside |

Table S1: (continued) LC.MS analysis for extract different organs of chamomile.

| **Whole plant** | | | **Aerial parts** | | | **Root** | | |  | | | | | |
| --- | --- | --- | --- | --- | --- | --- | --- | --- | --- | --- | --- | --- | --- | --- |
| **Rt (min)** | **Area %** | **Existence** | **Rt (min)** | **Area %** | **Existence** | **Rt (min)** | **Area %** | **Existence** | **MW** | **m.z [M-H]^+^** | **m.z [M- H]^-^** | **Type** | **Formula** | **Probable Compounds** |
|  |  |  | 1.583 | 0.78 | * |  |  |  | 408 |  | 407 | Phenolic compounds | (C27H52O2) | Methyl-19-hexacosenoate |
|  |  |  | 7.743 | 4.69 | * |  |  |  | 448 448  448.37  285 , 175  448.4  448.4 |  | 447 | Flavonoid | (C21H20O11) | Luteolin-7-O-glucoside or  Kaempferol-3-O-glucosid or  Luteolin glucoside or Luteolin hexoside or Quercetin-3-O-rhamnoside or  Quercetrin (quercetin-3-o-rhamonoside) |
|  |  |  | 1.583 | 0.78 | * |  |  |  | 408 |  | 407 | Phenolic compounds | (C27H52O2) | Methyl-19-hexacosenoate |
|  |  |  | 7.743 | 4.69 | * |  |  |  | 448 448  448.37  285 , 175  448.4  448.4 |  | 447 | Flavonoid | (C21H20O11) | Luteolin-7-O-glucoside or  Kaempferol-3-O-glucosid or  Luteolin glucoside or Luteolin hexoside or Quercetin-3-O-rhamnoside or  Quercetrin(quercetin-3-o-rhamonoside) |
|  |  |  | 1.583 | 1.56 | * |  |  |  | 486.7 |  | 485 | Triterpenoids | (C30H46O5) | Melilotigenin |
|  |  |  | 7.485 | 0.78 | * |  |  |  | 594 |  | 593 | Flavonoid | (C27H30O15)  (C27H30O15) (C27H30O15) (C27H30O15) | Leteolin-7-O-rutinoside or Apigenin-7-O- caffeylglucoside or Apigenin-6,8-dic-glucoside or Kaempferol-3-O-rutinoside |
|  |  |  | 7.99 | 3.91 | * |  |  |  | 610 |  | 609 | Flavonoid | (C27H30O16) | Rutin or  Quercetin-3-O- rhamnosylgalactoside or Quercetin-3-O-rutinoside or Rutin trihydrate |
|  |  |  | 1.583 | 1.95 | * |  |  |  | 640.5 |  | 639 | Flavonoid | (C27H28O18) | Quercetin-3-O-glucuronide-7-O-galactosid |
|  |  |  | 7.990 | 3.91 | * |  |  |  | 711.2114  711.2152 |  | 711 | (Flavonoid) (Saponin) | (C32H39O18)  (C32H39O1) | Not identified |
|  |  |  | 7.990 | 5.08 | * |  |  |  | 714 |  | 713 | - | (C42H49O10) | Not identified |
|  |  |  | 1.583 | 1.95 | * |  |  |  | 726 |  | 725 | Flavonoid | (C22H23O12) | Patuletin-3-O-manolylrobinobioside |
|  |  |  | 1.583 | 1.95 | * |  |  |  | - |  | 842 | - | - | Not identified |
| 10.891 | 4.43 | * | 17.703 | 1.69 | * |  |  |  | - | 86 |  | Laminene | - | Not identified |

Table S1: (continued) LC.MS analysis for extract different organs of chamomile.

| **Whole plant** | | | **Aerial parts** | | | **Root** | | |  | | | | | |
| --- | --- | --- | --- | --- | --- | --- | --- | --- | --- | --- | --- | --- | --- | --- |
| **Rt (min)** | **Area %** | **Existence** | **Rt (min)** | **Area %** | **Existence** | **Rt (min)** | **Area %** | **Existence** | **MW** | **m.z [M-H]^+^** | **m.z [M- H]^-^** | **Type** | **Formula** | **Probable Compounds** |
|  |  |  | 16.411 | 1.41 | * |  |  |  | - | 87 |  | - | - | Not identified |
|  |  |  | 0.658 | 1.69 | * |  |  |  | 100.158 | 101 |  | Flavenone | (C30H50O) | Cycloeucalenol |
|  |  |  | 1.668 | 8.45 | * |  |  |  | - | 104 |  | Laminene | - | Not identified |
|  |  |  | 18.572 | 3.38 | * |  |  |  | 108.09--108.02 | 109 |  | quinone | (C6H4O2) | 1,2-Benzoquinone |
|  |  |  | 18.572 | 1.13 | * |  |  |  | 114.19 | 114 |  | Alcohol | (C7H14O) | 3‑Methyl-cyclohexanol |
| 6.871 | 5.45 | * | 2.725 | 6.76 | * |  |  |  | 120.15 | 120 |  | Flavenone | (C8H8O) | 4-Vinylphenol |
|  |  |  | 8.352 | 0.56 | * |  |  |  | 142.24 | 143 |  | Flavenone | (C9H18O) | n-Nonanal |
| 3.657 | 5.64 | * | 1.668 | 6.20 | * |  |  |  | 144.25 | 145 |  | Flavenone | (C9H20O) | n-Nonanol |
|  |  |  | 18.572 | 0.00 | * |  |  |  | 172.26 | 173 |  | Flavenone | (C10H20O2) | Ethyl octanoate |
|  |  |  | 7.636 | 2.25 | * |  |  |  | 178 | 178 |  | Cumarine derivative | (C10H8O3) | Herniarin |
|  |  |  | 16.411 | 4.23 | * |  |  |  | 184.32--184.277 | 184 |  | Sesquiterpenes | (C12H24O) (C14H16) | 3-Dodecanone or Chamazulene |
| 11.425 | 4.91 | * | 7.636 | 8.73 | * |  |  |  | 196.29 | 196 |  | Sesquiterpenes | (C12H20O2) (C12H20O2) | Bornyl acetate or Neryl acetate |
|  |  |  | 8.646 | 2.82 | * |  |  |  | 198.39 | 198 |  | Alkaloid | (C11H12O3) | Myristicin |
|  |  |  | 16.246 | 2.82 | * |  |  |  | 200.23 | 200 |  | Phenolic compounds | (C13H12O2) (C13H12O2) (C13H12O2) | Cis-en-yn-Dicycloether or Trans-en-yn-Dicycloether or En-yn-Dicycloether |
|  |  |  | 15.447 | 1.41 | * |  |  |  | 204.35 | 204 |  | (Sesquiterpenes) (Alkaloid) | (C15H24)  (C15H24) (C15H26O) | Daucene or  β-Elemene or Germacrene-D or β-Selinene or α-Muurolene or β-Bisabolene or cis-β- farnesene |
|  |  |  | 18.572 | 1.41 | * |  |  |  | 206.37 | 206 |  | Sesquiterpenes | (C15H24) (C15H24) | Alpha‑Amorphene or Caryophyllene |

Table S1: (continued) LC.MS analysis for extract different organs of chamomile.

| **Whole plant** | | | **Aerial parts** | | | **Root** | | |  | | | | | |
| --- | --- | --- | --- | --- | --- | --- | --- | --- | --- | --- | --- | --- | --- | --- |
| **Rt (min)** | **Area %** | **Existence** | **Rt (min)** | **Area %** | **Existence** | **Rt (min)** | **Area %** | **Existence** | **MW** | **m.z [M-H]^+^** | **m.z [M- H]^-^** | **Type** | **Formula** | **Probable Compounds** |
|  |  |  | 16.246 | 2.82 | * |  |  |  | 222.366 | 222 |  | Sesquiterpenes | (C15H26O) (C15H26O) (C15H24) (C15H26O) (C15H26O) (C15H26O) (C15H26O) | Alpha-bisabolol or epi-α-bisabolol or farnesol or β-Bisabolol or β-Eudesmol(+)^-^ or Valeranone or  γ-Eudesmol |
|  |  |  | 18.572 | 0.56 | * |  |  |  | 224.255 | 224 |  | Flavenone | - | Not identified |
| 0.876 | 5.45 | * | 18.572 | 0.56 | * |  |  |  | 228.76 | 228 |  | Sesquiterpenes | (C15H26O) | Nerolidol |
|  |  |  | 8.352 | 3.38 | * |  |  |  | 340 | 341 |  | - | (C25H27O4) | Not identified |
| 10.871 | 5.45 | * | 7.636 | 1.41 | * |  |  |  | 375 | 375 |  | Flavenone | (C19H18O8) | Dihydroxy-tetramethoxy Flavone |
|  |  |  | 8.352 | 0.56 | * |  |  |  | 414---414.7 | 414 |  | sterol | (C16H29O12) | Unknown (Phytosterol) |
| 12.433 | 3.69 | * | 8.352 | 1.41 | * |  |  |  | - | 681 |  | - | - | Not identified |
| 12.433 | 7.62 | * |  |  |  |  |  |  | (133, 105, 89, 77 ) 162.142 |  | 161 | Cumarine | (C9H6O3) | Umbelliferone or Umbelliferone aglycone |
| 13.239 | 1.23 | * |  |  |  |  |  |  |  |  | 162 | - | - | Not identified |
| 9.945 | 6.64 | * |  |  |  |  |  |  | 164.16 |  | 163 | Phenolic compounds | (C9H8O3) | M-coumaric acid or P-Coumaric acid or Hydroxycinnamic acid |
| 12.433 | 0.74 | * |  |  |  |  |  |  | 205.21 |  | 204 | Indole derivative | (C11H11NO3) | Indolelactic acid |
| 12.433 | 0.49 | * |  |  |  |  |  |  | - |  | 223 | - | - | Not identified |
| 13.561 | 5.66 | * |  |  |  |  |  |  | - |  | 224 | - | - | Not identified |
| 13.561 | 5.66 | * |  |  |  |  |  |  | 286 |  | 285 | Flavonol | (C15H10O6) (C16H12O5) (C15H10O6) | Kampherol or Apigenin-4-methyl or Luteolin |
| 12.433 | 0.74 | * |  |  |  |  |  |  | 286 |  | 286 | Flavonol | (C15H10O6) | Kampherol |
| 12.433 | 3.69 | * |  |  |  |  |  |  | 304.25 |  | 303 | Flavonoid | (C15H12O7) | Taxifoline |
| 10.277 | 7.38 | * |  |  |  |  |  |  | 316 |  | 315 | (Phenolic compounds)  ( Flavonoid) | (C18H36O4) | Dihydroxy-octadecanoic acid or Isorhamnetin |

Table S1: (continued) LC.MS analysis for extract different organs of chamomile.

| **Whole plant** | | | **Aerial parts** | | | **Root** | | |  | | | | | |
| --- | --- | --- | --- | --- | --- | --- | --- | --- | --- | --- | --- | --- | --- | --- |
| **Rt (min)** | **Area %** | **Existence** | **Rt (min)** | **Area %** | **Existence** | **Rt (min)** | **Area %** | **Existence** | **MW** | **m.z [M-H]^+^** | **m.z [M- H]^-^** | **Type** | **Formula** | **Probable Compounds** |
| 10.478 | 3.69 | * |  |  |  |  |  |  | 341 |  | 340 | Alkaloid | (C20H23NO4) | Norargemonine |
| 12.433 | 0.74 | * |  |  |  |  |  |  |  |  | 353 | Phenolic compounds | (C16H18O9) | Chlorogenic acid or Cryptochlorogenic acid or Neochlorogenic acid or 4-O-caffeoylquinic acid or Caffeoylquinic acid or 1-Caffeyolquinic acid or 3-Caffeoylquinic acid or Chlorogenic acid or 4-Caffeoylquinic acid |
| 12.433 | 0.42 | * |  |  |  |  |  |  | 412.69 |  | 411 | Steroids | (C29H48O) | Stigmasterol |
| 12.433 | 0.98 | * |  |  |  |  |  |  | - |  | 412 | - | - | Not identified |
| 20.936 | 3.69 | * |  |  |  |  |  |  | - |  | 413 | - | (C16H29O12) | Not identified |
| 12.433 | 3.69 | * |  |  |  |  |  |  | 508 |  | 507 | Phenolic compounds | (C26H28O11) | Tetrahydroxy-dimethooxy-7- O-glucoside |
| 12.433 | 0.49 | * |  |  |  |  |  |  | 518 (323, 281, 193, 179, 161, 134) |  | 517 | Flavonoid | (C9H8O) (C25H24O12) | Ap-7- O-(6"-malonyl-Glc) or Ferulic acid or  Caffeic acid derivative or 1,5-Dicaffeoylquinic acid |
| 20.342 | 4.18 | * |  |  |  |  |  |  | 536.8 |  | 535 | (Alcohol) (Carotenoids) | (C37H76O) (C40H56) | 1-Heptatriacotanol or Betacarotene |
| 20.342 | 1.23 | * |  |  |  |  |  |  | 552.8 |  | 551 | - | - | Not identified |
| 12.433 | 0.74 | * |  |  |  |  |  |  | - |  | 693 | - | - | Not identified |
| 12.433 | 0.74 | * |  |  |  |  |  |  | 912 |  | 911 | Triterpene | - | Hederagenin glycoside |
| 13.239 | 7.38 | * |  |  |  |  |  |  | - |  | 1213 | - | - | Not identified |
| 12.433 | 3.44 | * |  |  |  |  |  |  | - |  | 1268 | - | - | Not identified |
| 0.876 | 2.55 | * |  |  |  |  |  |  | - | 84 |  | - | - | Not identified |
| 2.99 | 5.64 | * |  |  |  |  |  |  | 136.23 | 136 |  | Monoterpene | (C10H16) | Tricyclene or  Thujene-α or Camphene or  Myrcene or α-Phellandrene or α-Terpinene or  Limonene or γ-Terpinene or α-Terpinolene or Camphene |
| 19.858 | 5.64 | * |  |  |  |  |  |  | 144.25 144 | 144 |  | (Flavenone) (Alcohol) | (C9H20O) (C8H16O2) | n-Nonanol or 2-Propyltetrahydropyran-3-ol |
| 9.904 | 1.27 | * |  |  |  |  |  |  | 146.14 | 147 |  | Coumarin | - | Not identified |

Table S1: (continued) LC.MS analysis for extract different organs of chamomile.

| **Whole plant** | | | **Aerial parts** | | | **Root** | | |  | | | | | |
| --- | --- | --- | --- | --- | --- | --- | --- | --- | --- | --- | --- | --- | --- | --- |
| **Rt (min)** | **Area %** | **Existence** | **Rt (min)** | **Area %** | **Existence** | **Rt (min)** | **Area %** | **Existence** | **MW** | **m.z [M-H]^+^** | **m.z [M- H]^-^** | **Type** | **Formula** | **Probable Compounds** |
| 19.858 | 3.27 | * |  |  |  |  |  |  | 152.23 | 152 |  | Monoterpene | (C10H16O) | Artemisia ketone or Camphor or α -Bisabolol oxide A |
| 11.425 | 0.91 | * |  |  |  |  |  |  | 154.25 | 154 |  | Monoterpene | [(C10H18O](https://pubchem.ncbi.nlm.nih.gov/#query=C10H18O)) | 1,8-Cineole |
| 0.876 | 3.45 | * |  |  |  |  |  |  | 168 | 168 |  | Phenolic compounds | (C10H16O) (C8H8O4) | Trans-4-methoxy thujane or vanillic acid |
| 9.904 | 5.45 | * |  |  |  |  |  |  | 188.21 | 189 |  | Amine | (C11H19N) | Tricyclo[4.3.1.1(3,8)] undecan-1-amine |
| 19.425 | 5.45 | * |  |  |  |  |  |  | - | 202 |  | - | - | Not identified |
| 18.78 | 5.64 | * |  |  |  |  |  |  | 280.4 | 280 |  | Phenolic compounds | (C18H32O2) | Linoleic acid or 9,12-Octadecadienoic acid |
| 18.78 | 2.91 | * |  |  |  |  |  |  | 464.38 464 464 464 464 464.0955 464.0955 (301, 151, 121) | 465 |  | Flavonoid | (C21H20O12) | Hyperoside (quercetin-3-o-galactoside) or Quercetin galactoside or PentahydroxyFlavone-7-O-hexoside or Quercetin-7-O-glucoside or  PentahydroxyFlavone-7-O-hexoside or Kaempferol-3-O-glucuronide or  Isoquercitrin or Quercetin hexoside |
| 19.858 | 5.64 | * |  |  |  |  |  |  | 518 | 519 |  | Flavonoid | (C24H22O13) | Ap-7-O-(6"-malonyl-Glc)  or Apigenin-7-O- malonylglucoside |

Table S2. Mean, standard deviation, t and p-values of wheat and flixweed experiments.

| P-value | t-value | Flixweed (n=27) | Wheat (n=27) | Parameters |
| --- | --- | --- | --- | --- |
|  |  | Mean±SD | Mean±SD |  |
| <0001* | 7.13 | 68.21±25.6 | 111.9±19.0 | Germination rate  (% of control) |
| 0.7061 | 0.38 | 92.58±15.7 | 94.01±11.6 | Germination percentage  (% of control) |
| 0.0015* | 3.35 | 127.9±28.2 | 160.0±41.2 | Hydrogen peroxide  (% of control) |
| <0001* | -6.90 | 377.9±173 | 145.2±29.2 | Malondialdehyde  (% of control) |
| 0.0196* | -2.41 | 787.6±765 | 412.2±267 | Proline content  (% of control) |
| 0.0042* | -3.00 | 412.4±193 | 278.1±130 | Relative Evans blue uptake  (% of control) |
| <0001* | 7.23 | 56.43±17.7 | 89.94±14.4 | Radicle length  (% of control) |
| 0.0038* | 3.03 | 67.95±11.8 | 78.73±14.2 | Plumule length  (% of control) |
| <0001* | 5.17 | 59.27±15.9 | 81.72±16.1 | Seedling length  (% of control) |
| 0.0102* | 2.67 | 55.27±16.6 | 66.88±15.4 | Seedling dry weight  (% of control) |
| 0.0006* | 3.64 | 56.32±20.3 | 78.38±24.0 | Seedling vigor index  (% of control) |

* Indicates a significant difference between the two group.

Table S2. Mean, standard deviation, t and p-values of wheat and flixweed experiments.

| P-value | t-value | Flixweed (n=27) | | Wheat (n=27) | | Parameters |
| --- | --- | --- | --- | --- | --- | --- |
|  |  | Std. Deviation | Mean | Std. Deviation | Mean |  |
| <0001* | 7.13 | 25.58 | 68.21 | 19.00 | 111.9 | Germination rate  (% of control) |
| 0.7061 | 0.38 | 15.71 | 92.58 | 11.67 | 94.01 | Germination percentage  (% of control) |
| 0.0015* | 3.35 | 28.20 | 127.9 | 41.17 | 160.0 | Hydrogen peroxide  (% of control) |
| <0001* | -6.90 | 172.7 | 377.9 | 29.23 | 145.2 | Malondialdehyde  (% of control) |
| 0.0196* | -2.41 | 764.8 | 787.6 | 266.7 | 412.2 | Proline content  (% of control) |
| 0.0042* | -3.00 | 193.2 | 412.4 | 130.0 | 278.1 | Relative Evans blue uptake  (% of control) |
| <0001* | 7.23 | 17.67 | 56.43 | 16.35 | 89.94 | Radicle length  (% of control) |
| 0.0038* | 3.03 | 11.79 | 67.95 | 14.24 | 78.73 | Plumule length  (% of control) |
| <0001* | 5.17 | 15.87 | 59.27 | 16.06 | 81.72 | Seedling length  (% of control) |
| 0.0102* | 2.67 | 16.577 | 55.27 | 15.42 | 66.88 | Seedling dry weight  (% of control) |
| 0.0006* | 3.64 | 20.27 | 56.32 | 24.04 | 78.38 | Seedling vigor index  (% of control) |

(*) Indicates a significant difference between the two groups.
